# Optical Imaging of Human Cone Photoreceptors Directly Following the Capture of Light

**DOI:** 10.1371/journal.pone.0079251

**Published:** 2013-11-15

**Authors:** Phillip Bedggood, Andrew Metha

**Affiliations:** Department of Optometry and Vision Sciences, University of Melbourne, Parkville, Victoria, Australia; Dalhousie University, Canada

## Abstract

Capture of light in the photoreceptor outer segment initiates a cascade of chemical events that inhibit neurotransmitter release, ultimately resulting in vision. The massed response of the photoreceptor population can be measured non-invasively by electrical recordings, but responses from individual cells cannot be measured without dissecting the retina. Here we used optical imaging to observe individual human cones in the living eye as they underwent bleaching of photopigment and associated phototransduction. The retina was simultaneously stimulated and observed with high intensity visible light at 1 kHz, using adaptive optics. There was marked variability between individual cones in both photosensitivity and pigment optical density, challenging the conventional assumption that photoreceptors act as identical subunits (coefficient of variation in rate of photoisomerization = 23%). There was also a pronounced inverse correlation between these two parameters (p<10^−7^); the temporal evolution of image statistics revealed this to be a dynamic relationship, with cone waveguiding efficiency beginning a dramatic increase within 3 ms of light onset. Beginning as early as 2 ms after light onset and including half of cells by ∼7 ms, cone intensity showed reversals characteristic of interference phenomena, with greater delays in reversal corresponding to cones with more photopigment (p<10^−3^). The timing of these changes is argued to best correspond with either the cessation of dark current, or to related events such as changes in intracellular cGMP. Cone intensity also showed fluctuations of high frequency (332±25 Hz) and low amplitude (3.0±0.85%). Other groups have shown similar fluctuations that were directly evoked by light; if this corresponds to the same phenomenon, we propose that the amplitude of fluctuation may be increased by the use of a bright flash followed by a brief pause, to allow recovery of cone circulating current.

## Introduction

The first stage of seeing is mediated by the photoreceptors, which capture photons and adjust their rate of neurotransmitter release accordingly. The series of chemical reactions that comprise this process is known as the phototransduction cascade. The various stages of the cascade have previously been elucidated through careful recordings of the current flowing through individual photoreceptors in isolated retina [1], [2], and of the potential difference generated at the front of the eye when the photoreceptor population responds in unison (electroretinogram, or ERG [3]). Key features of the cascade have also been revealed optically, analyzing light-evoked scatter in the infra-red in isolated preparations of photoreceptor outer segment fragments [4]–[6].

Such work shows how photoreceptors respond to light in general, but does not facilitate the study of individual photoreceptor responses *in vivo*. The ability to compare functional signals from individual photoreceptors would facilitate investigations into the fundamental arrangement of the human visual system, and may provide more sensitive measures by which retinal diseases can be detected and treated. Here, we have imaged individual cone photoreceptors at high speed during bleaching of their photopigment and ensuing phototransduction. The results highlight baseline differences between cones in the healthy eye and reveal rapid intensity changes linked to the bleaching of photopigment, to alterations in cone waveguiding efficiency, and to the metabolic consequences of phototransduction.

### Overview of the Phototransduction Cascade

Visual pigment consists of a membrane-bound G-protein coupled receptor (opsin) whose seven transmembrane helices form a binding pocket for the chromophore *11-cis* retinal [7]. Upon absorption of a photon, the chromophore isomerizes to *all-trans* retinal over a period of picoseconds or less [8]. The *all-trans* isomer is unstable and undergoes serial decay; the first product that is somewhat stable on the millisecond timescale is Metarhodopsin I, which has peak absorptance blue-shifted by ∼20 nm [9] (NB: ‘Metarhodopsin’ historically refers to the compound found in rods, but we will follow the literature and use the same name for the corresponding compound in the cones).

Metarhodopsin II is formed upon deprotonation of the Schiff base linking the chromophore to the opsin molecule. This is an essentially non-reversible process that occurs exponentially, with time constant ∼0.25–0.50 ms [7]. Since the peak absorptance of Metarhodopsin II is blue-shifted by ∼120 nm compared with the original pigment, it appears colourless or ‘bleached’ at visible wavelengths. This change is measurable *in* *vivo* by quantifying the increase in light reflected from the retina compared with the dark-adapted state [10].

Metarhodopsin II acts as an enzyme, catalyzing activation of the G-protein transducin [7]. Transducin couples very rapidly with the enzyme phosphodiesterase (PDE), forming a complex which catalyzes the hydrolysis of cyclic guanosine monophosphate (cGMP). Before hydrolysis, cGMP is responsible for holding open sodium influx channels in the outer segment that maintain the cell in a relatively depolarized state whilst in the dark, such that a “dark current” flows through the cell. This depolarized state allows voltage-gated calcium influx channels to remain open. The resulting high concentration of intracellular calcium causes constant release of the neurotransmitter glutamate in the dark. In the light then, the hydrolysis of cGMP closes the sodium channels, causing the cell to hyperpolarize. This closes calcium channels and so inhibits glutamate release [7].

### Adaptive Optics Imaging of Cone photoreceptor Fluctuations

Adaptive optics corrects the wavefront aberrations of the eye in order to allow diffraction-limited imaging of the retina *in* *vivo*, allowing individual photoreceptors to be resolved in humans [11]. Using adaptive optics, baseline intensity fluctuations in rods and cones have been tracked over time scales of minutes and hours [12]–[14], and light-evoked fluctuations have been tracked on the scale of fractions of a second to milliseconds [15]–[18]. A large component of observed variability is thought to result from the multiple sites of reflectance along the length of the cone outer segment, which produce a “biological interferometer” that is a sensitive gauge of changes in either cell length, refractive index or scatter within the outer segment [13], [15], [17].

The fastest temporal measurements to date have been made using long coherence infra-red light to image the cone mosaic at 192 fps [17]. After stimulation with a single flash of visible light, high frequency intensity fluctuations began ∼5–10 ms after light onset and lasted for ∼300–400 ms. This signal demonstrated random starting phase and required coherent light, confirming the interference-driven mechanism described above [17]. The rapid onset and broad temporal spectrum of intensity fluctuations has been argued to indicate a non-linear process, ruling out changes in the concentration of activated opsin, transducin or PDE [17]. The time course of the single-flash response, which lasts up to several hundred milliseconds, agrees well with electrophysiological data from single cones [2], and thus the most likely explanation relates either to the cone circulating current directly, or to associated factors such as membrane polarization or levels of cGMP. It is not known whether these processes act to change refractive index, or to increase scatter, or to increase the outer segment length through osmotic activity [17].

We have previously imaged the human cone mosaic at 12 fps during stimulation with high intensity bleaching light, to observe the time course of bleaching and of slower light-evoked optical signals [15]. We were able to demonstrate marked variability in the photosensitivity and in the amount of visual pigment between nominally identical cones, and found an inverse association between these parameters. We also observed that, despite the relatively short coherence length of our imaging light (7 µm as described previously [15]), the responses of many cones were dominated by interference-driven effects. This is much shorter than the length of the cone outer segment, suggesting that significant scatter can arise within the outer segment, presumably as a result of metabolic processes within the cell. Evidence of pronounced interference-driven fluctuations under short coherence illumination are apparent in other work also [12], [14], [16], [18].

The present work aimed to divorce the exponential photo-bleaching response from interference-driven fluctuations in intensity, by imaging at much faster frame rate (1000 fps) than the 12 fps that was possible previously [15]. Separating these components of the optical response is expected to reveal more about the properties of each, and also about the differences in these properties between neighboring cones.

## Methods

### Subject Selection and Area Imaged

This project was carried out in accordance with the principles expressed in the Declaration of Helsinki, and approved by the University of Melbourne Human Ethics Committee. Written informed consent was obtained from all subjects before testing. Pilot measurements were initially conducted on 8 young, healthy volunteers (Optometry students, and the first author). As we observed previously [15], useful data requires reproducible fixation and head position, highly resolvable cones close to the centre of the fovea, and a wide foveal avascular zone. The requirements on reproducibility of fixation are especially important in the present work due to the limited vertical extent of the area imaged (explained below). For these reasons we selected only a single subject to undergo the full experimental protocol. This subject, who is also the first author, was a healthy 29-year-old male with emmetropic refraction and normal ocular health.

**Figure 1:**
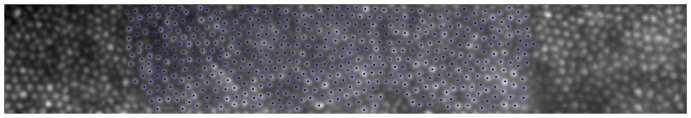
Average cone image for all sequences, used as master image for cone labeling. Imaged area is ∼1° inferior to fixation, and is within the foveal avascular zone. Labeled cone centres are marked in blue (n = 510). The average of corresponding frames in all sequences is presented as a pseudo-sequence in Media S1, which is cropped so that only pixels that were illuminated in every sequence are shown. The limited vertical extent of the field facilitates high frame rate acquisition with the sCMOS hardware.

We imaged an area in the left eye 1° inferior to fixation, as depicted in Figure 1. Both eyes were dark-adapted for 6 minutes before adaptive optics corrected image sequences were acquired (detailed below). This procedure was repeated 16 times over the course of two days. We rejected 8 sequences in total, leaving 8 sequences for analysis. The reason for the high rate of rejection is a result of a) the small vertical extent of the field of view, required for high frame rate acquisition with the sCMOS hardware. In 4 sequences the subject evidently used a second locus of fixation displaced ∼28 µm in the vertical direction (or 70% of the vertical field of view); and b) in 4 sequences the image quality was noticeably inferior, which we believe results from the head not being repositioned to coincide sufficiently with the peak of the Stiles-Crawford function. It is not possible to test this alignment by taking a pilot image beforehand, since the retina must remain dark-adapted. The other subjects that we attempted showed substantially less consistency, either in fixation and/or in image quality.

The 8 acceptable sequences for this subject were registered with one another as described previously [15], and an average image generated from these (Figure 1). Cone positions were labeled manually on this image, followed by automatic fine-tuning of labeled positions to find the centre of gravity of each cone [15]. 510 cones were labeled in this fashion; their positions are plotted overlaying the cone image in Figure 1.

### Adaptive Optics Imaging

We used a flood-illuminated adaptive optics opthalmoscope. Compared to our previous [15] system for photoreceptor imaging, the current [19] system is improved in design in terms of light throughput (allowing shorter exposures) and adaptive optics correction quality.

The wavefront beacon is an 835-nm superluminescent diode (Hamamatsu, Hamamatsu City, Japan). The wavefront sensor is a Hartmann-Shack with lenslets of 0.4-mm pitch and 24-mm focal length (Adaptive Optics Associates, Cambridge, MA), coupled to a charge-coupled device camera (Pike, Allied Vision Technologies, Stadtroda, Germany). The deformable mirror is a HiSpeed DM97-15 (Alpao, Montbonnot St. Martin, France) with 13.5 mm diameter, which corresponds to 7.6 mm in the pupil plane. Adaptive optics correction operated in closed loop at 20 Hz using custom Matlab software (Mathworks, Natick, MA). When the measured root mean square (RMS) wavefront error became sufficiently low (typically <0.06 µm over a 7 mm pupil), a TTL pulse was sent to the imaging camera, an Andor Neo sCMOS (Andor Technology PLC, Belfast, UK). The camera was configured to expose all pixels simultaneously (“global” shutter) to 0.5 ms of imaging and stimulation light, with a frame rate of 1000 fps and 11-bit depth. This high speed is achieved by reducing the vertical extent of the imaged field to just 80 pixels (∼40 µm on the retina, or 0.14°, or ∼12 cones at this eccentricity), which allows the sCMOS architecture to spend less time reading out each frame. The horizontal extent is not restricted by this operation (illuminated field of view 1.25° diameter). As the camera begins each 0.5 ms exposure it outputs a TTL pulse lasting for the same duration, which was routed to the seed laser of our stimulation/imaging source, a 6W supercontinuum laser (Fianium Ltd., Southampton, UK). This causes the laser to turn on for 0.5 ms, delivering light that is essentially continuous during this time (pulses at 80,000 MHz). As described previously [15], an acousto-optic tunable filter (Crystal Technology, Palo Alto, CA) allows the selection of small wavebands for retinal imaging. We imaged over a band of 540–573 nm to maximize cone pigment optical density and minimize variability due to differences between L and M cones, while at the same time minimizing coherence length (7 µm FWHM in the cone outer segments, as measured with Michelson interferometry [15]). Light was passed through 32 m of 0.37 NA, 200 µm core diameter, step-index optical fiber (Thorlabs, Newton, NJ) to reduce coherence and associated image speckle. The fiber tip was made conjugate to the retina.

The system optics in the imaging arm are composed entirely of mirrors, save the final optic which is a lens. An “off the plane” design simultaneously minimizes pupil and retinal plane astigmatism [20], [21]. The mirrors were coated with a broadband dielectric coating to maximize throughput in the waveband used in the experiment. Together with the larger pupil and more sensitive camera compared with our previous work, this allowed reduction of exposure time from 3 ms to 0.5 ms.

Total corneal power was 0.62 mW. With 100 frames acquired in each sequence the energy delivered to the retina is 1.5 log units below the maximum permissible exposure (MPE) defined according to American National Standards Institute guidelines [22]. The corresponding retinal illuminance was calculated to be 8.97 log Td, giving 4.67×10^5^ Td.s of energy in each 0.5 ms pulse. Based on data from subjects studied by Rushton [10], this was predicted to bleach ∼21% of L/M cone photopigment in the first pulse (cf. 30% bleach measured in our actual data).

### Densitometry

Retinal densitometers traditionally employ a minimally visible, long wavelength reference light in order to quantify pigment optical density [10]. We previously argued that this approach is of dubious benefit on the scale of individual cones due to the dependence of baseline intensity on interference-driven factors, which are highly sensitive to wavelength differences [15]. We note also that the modal propagation of light through receptor waveguides can change sharply with wavelength; e.g. Snyder and Pask give a theoretical model in which bi-modal propagation at 555 nm transitions to uni-modal propagation above ∼650 nm [23]. The use of adjacent tissue (the cone interstitial spaces) as a reference is even less suitable, as this light is not waveguided at all.

Instead we followed our previous approach [15] and set our reference as the plateau intensity that is reached after bleaching of pigment is complete. This time-resolved approach was previously validated as a measure of pigment bleaching by the agreement of derived constants with published physiological data, by the absence of bleaching with 690 nm light, and by the absence of measurable bleaching when the retina was pre-bleached [15].

To determine the intensity of light reflected from each cone, we averaged a 3×3 pixel area centered on the cone. The details of the exponential bleaching model are as described previously [15]. The important parameters extracted from this model are the photosensitivity - i.e. the rate constant of the bleach (units of Td^−1^s^−1^), and pigment optical density (dimensionless).

**Figure 2:**
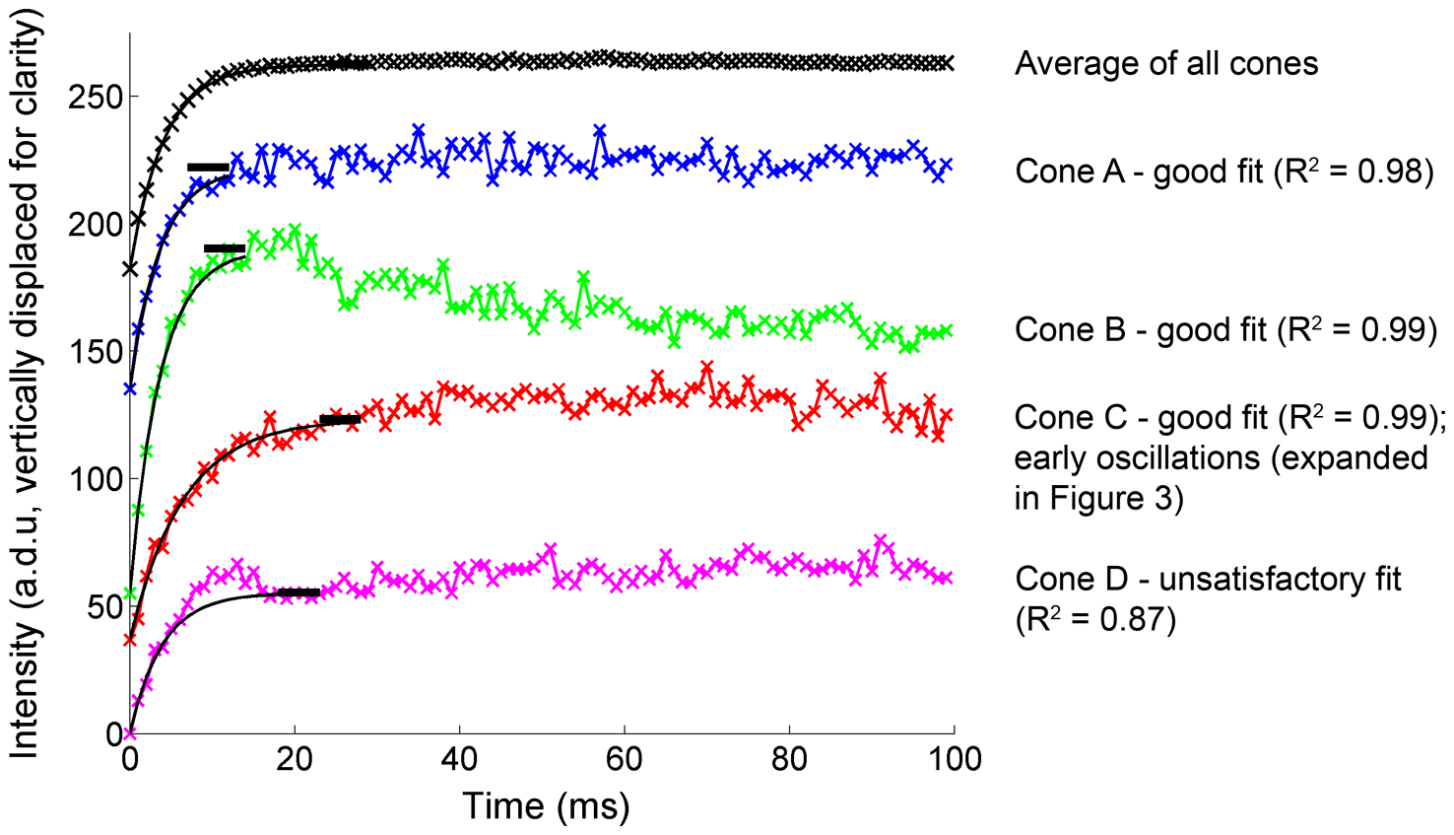
Representative data to illustrate curve fitting. Raw intensity from selected cones is shown (coloured crosses) together with the average of all cones (black crosses). Intensity traces are displaced vertically for clarity. After estimation of plateau intensity (black bars) from the raw data, an exponential fit was obtained (smooth black lines). The quality of fit was good (R^2^>0.95) for the average and for cones A, B and C, but unsatisfactory (R^2^<0.95) for cone D.

As we have observed previously, to extract reliable bleach parameters the influence of intensity fluctuations that are not a result of pigment bleaching must be minimized. Our current approach to mitigate this issue is depicted in Figure 2, which shows areas of the curve that were used in the fitting of sample data from one sequence. The average of all cones is shown (black symbols) together with the intensity of selected individual cones (colored symbols). Bleach parameters were estimated as follows:

#### Plateau intensity

When fitting an exponential curve of the form taken by our model, a large family of equivalent fits can often be found unless the plateau intensity is appropriately constrained. We fixed the plateau intensity by locating the 5 ms data window that showed the lowest coefficient of variation, considering only the first 30 ms of data to avoid the onset of intensity fluctuations. Taking the median intensity during this window provided an initial estimate of the plateau intensity. Since this does not represent the intensity as t→∞, we calculated the pigment density remaining at this time from the averaged cone data, and then elevated the individual estimate of plateau intensity accordingly (average elevation 0.9±1.0%). The black bars in Figure 2 demonstrate the plateau intensity derived in this way. This procedure overall was significantly more robust than our previous approach in which plateau intensity was left as a free parameter in the fit.

#### Optical density

After determination of plateau intensity, the absorptance was determined by the ratio between plateau intensity and intensity of the first frame. Absorptance was then converted to optical density [10].

#### Photosensitivity

This was the single free parameter for the exponential model fit, which was made to the first 6 frames (0–5 ms) of data.

As we observed in our previous experiment [15], some cones display fluctuations in intensity of sufficiently low latency and large amplitude that the fit quality of the simple exponential bleaching model is dramatically reduced. To avoid our analysis being unduly influenced by such cones we rejected those curve-fits with a coefficient of determination for the model (R^2^ goodness of fit measure) of less than 0.95. R^2^ was calculated in a conservative manner by including the intermediate data points that were not actually used in the fit (i.e. up to 30 ms, as opposed to 0–5 ms). It should be noted that we also repeated our analysis using a more typical curve fitting approach in which the fit was made to the first 20 ms of data, and in which all 3 parameters described above were allowed to vary without constraint. This did not appreciably alter the number of cones meeting the fit quality criterion, the distribution of their fitted parameters, or our conclusions regarding the statistical association between them.

### Fluctuations in Cone Intensity

**Figure 3:**
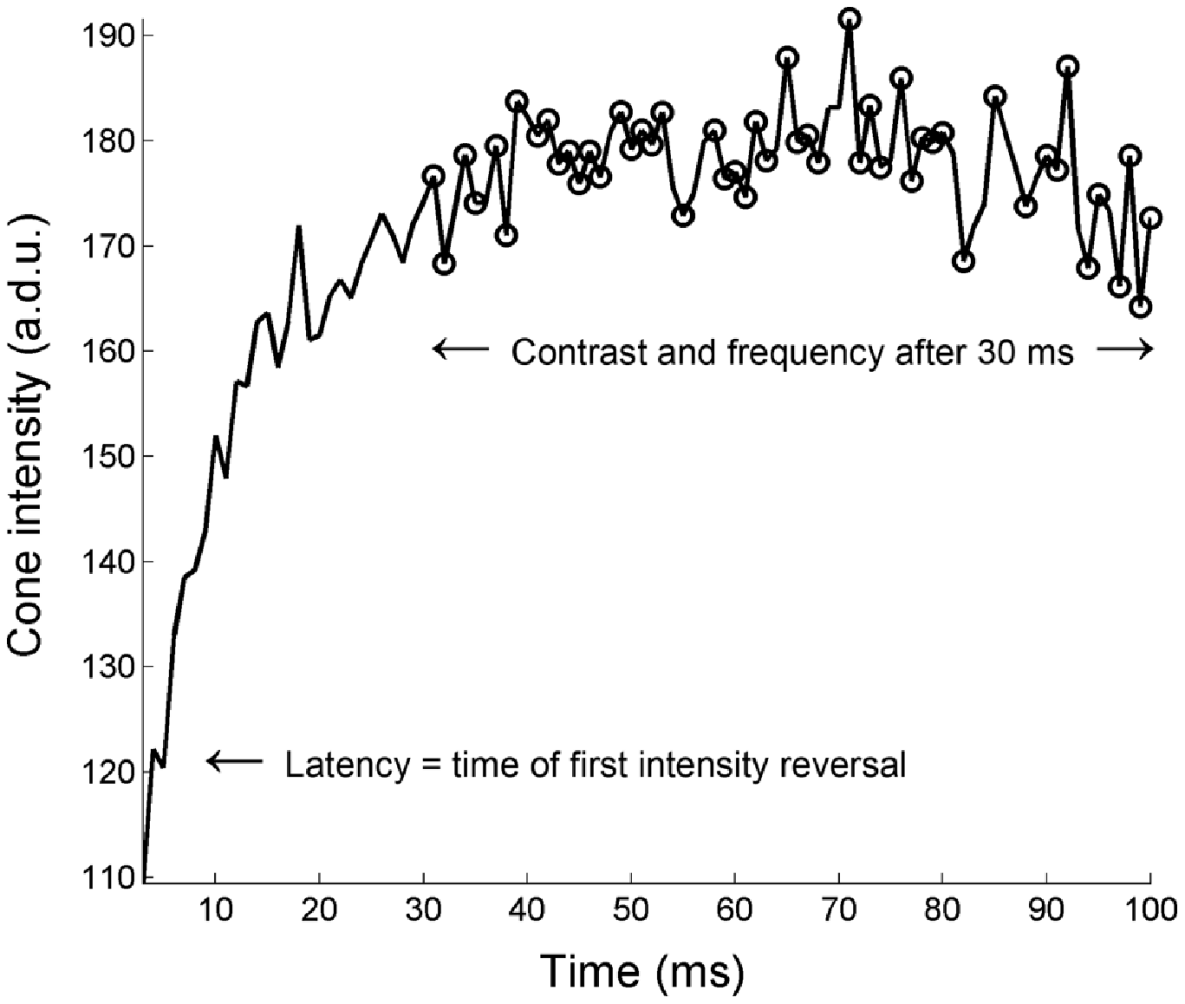
Representative data to illustrate measurement of intensity fluctuations. Latency was defined as the time at which a negative slope in the cone intensity trace was first detected. Contrast was determined by the coefficient of variation beyond 30) counting extrema beyond 30 ms, and b) wavelet analysis beyond 30 ms.

As is evident from the single cone responses in Figure 2, the expected increase in intensity due to pigment bleaching is accompanied by rapid fluctuations in cone intensity. Fluctuations were quantified in 3 ways, as illustrated in Figure 3:

#### Latency

The time at which each cone first showed a decrease in its intensity compared to the previous frame. Decreases in intensity cannot result from recovery of photopigment, which happens via the shortest known pathway over the course of minutes [24]. Decreases are instead thought to be caused by destructive interference, as discussed above. This metric is an overestimate of the true latency to fluctuation, since ∼50% of interference responses must in fact be constructive. Such events will not be easy to distinguish from intensity increases due to pigment bleaching, if they occur while significant pigment remains.

#### Frequency

This was analyzed both by automated counting of the numbers of successive peaks and troughs after the 30 ms mark (at which point all pigment should be well and truly bleached), as well as via wavelet analysis of the intensity trace for each cone.

#### Average contrast

Defined as the coefficient of variation (ratio of standard deviation to mean) over the 30–100 ms period. This metric was highly similar to the Michelson contrast between successive minima and maxima, which is another candidate metric for defining contrast of fluctuations.

### Limitations to Measurement of Cone Intensity Fluctuations

It is important to establish the degree to which we can distinguish fluctuations in cone intensity from quantal variations in photon capture (i.e. shot noise). Our calculated shot noise for a 3×3 pixel area, based on a gain of 20 electrons/a.d.u., quantal efficiency of 0.57, and raw average cone intensity in the first frame of ∼200 a.d.u., is ∼0.4%. This value will decrease somewhat during the bleach, as more photons reach the detector. It should also be noted that the effect of shot noise is independent of frequency, while the power spectrum of our intensity traces will be shown to exhibit strong frequency dependence.

It is also important to establish the temporal sampling limitations of our method. In the absence of a model that relates intensity fluctuations to the underlying biology, the temporal bandwidth of the optical phenomenon is unknown. This leaves some possibility of aliasing by frequencies beyond the Nyquist limit (>500 Hz). However we calculate that our 0.5 ms integration window should exclude such contributions above 1000 Hz (if of comparable contrast to the fluctuations that we measure here), and reduce the contrast of contributions in the 500–1000 Hz range. The broad frequency spectrum of our data suggests that any aliased contribution will itself be broad in spectrum, which should facilitate the reduction in contrast afforded by our 0.5 ms sampling window. Additionally we note that intensity fluctuations were not observed when imaging a static model eye with a paper “retina”, which rules out aliasing due to noise inherent in the imaging system itself. Any fluctuations due to accommodation or movements of the eye should produce changes that are in phase across all cones, which is not evidenced by our results. A biological (functional) signal in the range 500–1000 Hz is not ruled out, however.

In regards to the latency parameter, this is an “edge” rather than a frequency-based measurement. It is measurable to the precision dictated by our frame rate (1 ms), and is likely an overestimate as described above, but it should not be affected beyond this precision by any aliasing that is present.

Finally it is important to note that the intensities delivered to the eye were several orders of magnitude greater than physiological intensities that the retina is naturally exposed to. This is unlikely to affect the amount and rate of pigment bleach, but functional changes such as fluctuations and alterations to cone waveguiding could well be elicited in a different form to that encountered under natural conditions.

## Results

### Pigment Optical Density and Photosensitivity

Averaged across all sequences, cone double pass optical density was 0.273±0.040, photosensitivity was 2.54+/−0.49×10^−7^ Td^−1^s^−1^, and plateau intensity was 175±25.0 (arbitrary units). An average of 367 cones in each sequence contained sufficient data (i.e. did not become clipped by the narrow imaging field) for curve fitting.

On average 77% of cones achieved a fit quality R^2^>0.95, evaluated up to the first 30 ms of data as described above. Examples of accepted (A, B, and C) and rejected (D) cones were depicted in Figure 2 above. For comparison, in our previous work where a more lax criterion was adopted (R^2^ calculated for the first 7 frames only), only ∼28% of cones were accepted on average [15]. In other words the higher frame rate used here facilitates reliable estimates of bleaching parameters in a much larger proportion of cones. This is presumably because useful bleach information persists for a greater number of exposures under this paradigm, before being destroyed by fluctuations in cone intensity.

### Cone Intensity Fluctuations

**Figure 4:**
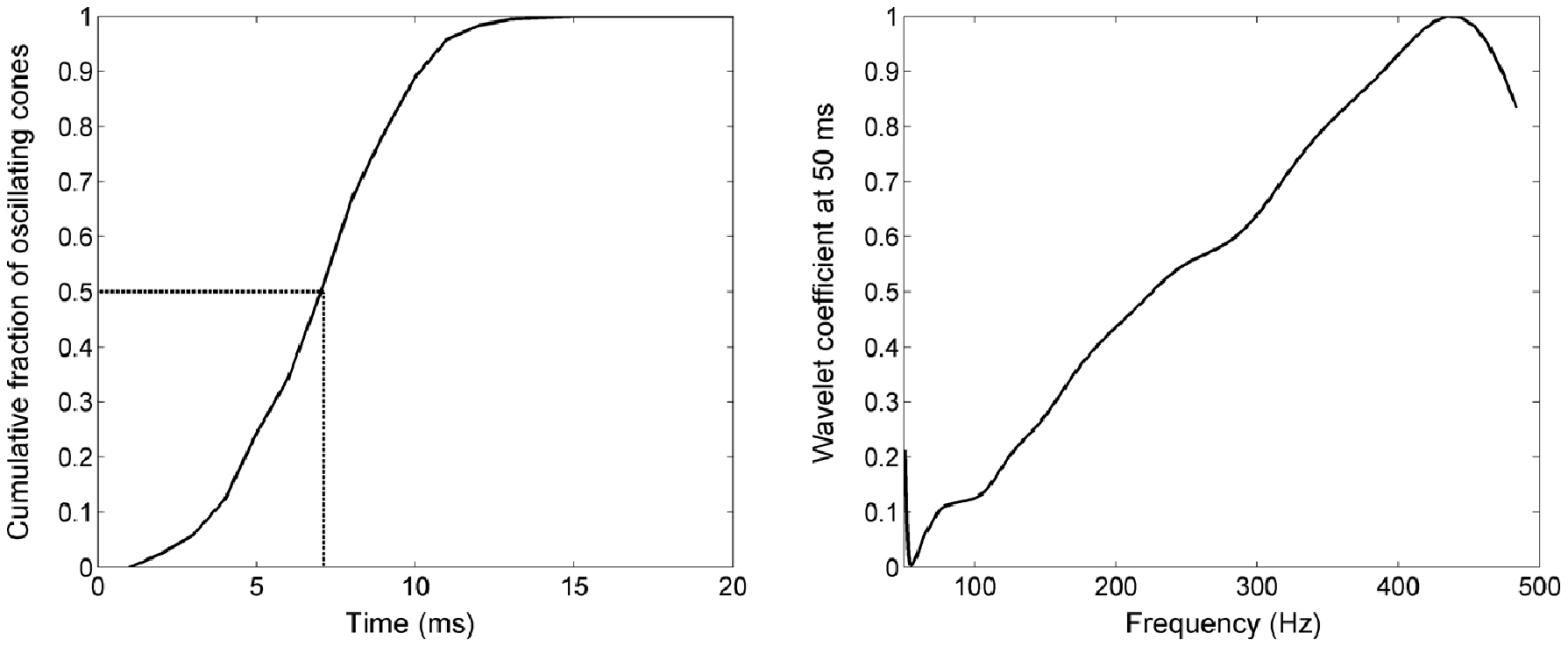
Fluctuation parameters for the cone population in one sequence. Left: Cumulative fraction of cones that showed detectable fluctuations, as a function of time. 50% of cones were observed to oscillate by ∼7 ms. Right: Average wavelet coefficient for the cone population in one sequence, at t = 50 ms (the time point at which valid sampling in frequency space is the widest). The linearity in frequency space is suggestive of a process that is non-linear with time.

The first reversal in cone intensity was seen in 1% of cones by 2 ms (the third frame), with 50% of cones showing the response by 7 ms. This sits squarely within the 5–10 ms onset reported for adaptive optics functional cone imaging in the past [17], and coincides with the latency of onset in changes to cone circulating current [25], [26]. 95% of our cones began to show the response by 12 ms. The cumulative distribution of cones showing the response as a function of time is shown in Figure 4 (left).

The contrast of intensity fluctuation was assessed from 30 ms onwards, and averaged 3.0±0.85%, with individual cone fluctuations as high as ∼12% in each sequence. This is slightly less than that reported in previous measurements at high frame rate [17] (∼4% average contrast, up to 20% in some cones); both measures are significantly less than the contrast measured at slower frame rates by both ourselves and others [12], [15], [18].

Frequency of fluctuation was also assessed from 30 ms onwards. Average frequency, determined by simply counting peaks and troughs in intensity, was 332±25 Hz. This agreed well with the dominant time-averaged frequency on wavelet analysis, which was 335±55 Hz. Wavelet analysis revealed distinct dominance at particular frequency bands in most individual cones, however, when averaged across all cones there were no common epochs in frequency or in time. The averaged frequency spectrum was in fact highly linear from ∼100–400 Hz, as shown in Figure 4 (right). Unsurprisingly then, none of the parameters extracted from wavelet analysis were correlated with the fitted bleach parameters or to the time of onset of intensity reversals.

As mentioned above, if the nature of the cone intensity fluctuations contain frequencies in the range ∼500–1000 Hz, aliasing may result which would mean that the true average frequency would be higher than reported above. If affected significantly by aliasing, the diffuse nature of the spectrum implies that the true signal would also be diffuse, leaving the qualitative nature of our conclusions regarding the spectrum unaltered.

### Correlation between Parameters

Pigment optical density was inversely correlated with photosensitivity in each sequence (median: p<10^−7^ and r = −0.32), confirming our previous observations [15]. Pigment optical density was positively correlated with the latency of noticeable fluctuations (median: p<10^−3^ and r = 0.21). Photosensitivity was correlated weakly with latency (median: p<0.05, r = −0.12), which is probably secondary to the stronger association between pigment and photosensitivity; this correlation in fact disappeared (p>0.4) when we employed the alternate curve fitting approach described above (all other correlations retained their significance with this approach, at the 95% level). Neither frequency nor contrast of fluctuations were significantly correlated with pre-bleach parameters.

We calculated Voronoi area [27] for each cone that was surrounded by other cones (i.e. not on the edge of the labeled area). Mean Voronoi area was 16.3±1.9 µm. This equates to a mean inter-cone distance at this eccentricity of ∼4.5 µm. In each sequence Voronoi area was correlated with optical density (median: p<0.05, r = −0.16), photosensitivity (median: p<10^−3^, r = 0.22), and contrast of fluctuations (median: p<0.05, r = −0.14). Voronoi area was also correlated with plateau intensity (median: p<10^−5^, r = 0.30). In other words, cones that occupied a larger area tended towards reduced optical density, more rapid bleaching, greater plateau intensitiy, and lower contrast fluctuations.

### Variability in Measured Parameters

The coefficient of variation in each measured parameter is shown in Table 1, locally across space (first column), across time (second column), and overall (final column). The local spatial measurements are the average coefficient of variation obtained for each cluster of 20 cones. This measure was derived to provide an estimate of the variability between neighbouring cones alone, as opposed to that between the whole population, since eccentricity-dependent differences in cone physiology are expected. The temporal measurements were assessed for cones which showed satisfactory fit quality in at least 5 sequences (264 cones). The overall estimate was derived by pooling all individual cone measurements across space and time.

**Table 1:** Average coefficient of variation across groups of measurements.

Parameter	Spatial (clusters of 20)	Temporal (> = 5 measurements)	Overall
Pigment density	0.132	0.113	0.168
Photosensitivity	0.169	0.149	0.248
Photoisomerization rate	0.171	0.154	0.226
Plateau Intensity	0.116	0.094	0.149
Fluctuation latency	0.303	0.289	0.330
Fluctuation frequency	0.074	0.072	0.075
Contrast	0.244	0.198	0.305
Voronoi area	0.223	–	0.117

Left: spatial variation, in which variability was calculated across each cluster of a cone and its 20 nearest neighbours. This was done to avoid the effects of eccentricity on cone physiology. Middle: temporal variation, in which variability was calculated across the same cone in time (minimum of 5 sequences of valid data required). Right: overall variation, in which spatial and temporal measurements were pooled.

## Discussion

### Variability in Rate of Photoisomerization between Cones

We have used time-resolved adaptive optics retinal densitometry to observe human foveal cone photoreceptors during the bleaching of visual pigment and ensuing phototransduction. Our measurements demonstrated marked differences in pigment optical density and in photosensitivity between neighbouring cones and within each cone from one cycle of bleaching and regeneration to the next. Is this source of noise likely to have a significant impact for vision? Although the high amount of noise generated by the cones in the dark is largely suppressed in the light, a significant amount of variability does in fact remain [28], [29]. The noise is thought to arise in the cone outer segment, but it has been difficult to say anything more concrete on its origins based on electrophysiological recordings [30].

The probability for photon capture can be predicted by the product of the photosensitivity and the optical density of pigment. Table 1 gives the coefficient of variation in this parameter (photoisomerization) as ∼23%, with all cone measurements pooled (cf. 17% and 25% for pigment density and photosensitivity, respectively). Alternately, the instantaneous, local (nearest 20 cones) coefficient of variation was ∼17%. If optical variations do in fact account for the majority of the noise in cone output, the electrical response should then show a similar coefficient of variation across the cone population (i.e. ∼17–23%).

Lamb and Simon measured electrical responses to photic stimulation in 30 cones from dissected turtle retina, and evaluation of their data shows a coefficient of variation of 22% in the maximum amplitude of the response (data from their Table 1 [28]). A somewhat larger value of 35% was found by Schnapf and others, who recorded from 26 cones in the macaque monkey (data also from their Table 1 [1]). Based on these comparisons it is plausible that anywhere from half to practically all of the noise in electrical response to light between neighbouring cones results from variations in optical parameters amongst the cones. Since the noise in the cone population is thought to explain almost all of the noise present in the photopic visual system [29], the optical variability between cones may indeed constitute a significant limitation for vision. This challenges the traditional assumption that cones of a common type produce identical responses, an assumption that has also been challenged recently on the basis of other adaptive optics imaging work [31].

### Statistical Distribution of Pigment Density and Photosensitivity

For computational neuroscience it is desirable to be able to simulate the response of the cone photoreceptor mosaic. Towards this end, could the predicted rate of photoisomerization for each cone be drawn from a simple Gaussian distribution? A Shapiro-Francia test [32] on the entire data set for all sequences showed that predicted rate of photoisomerization was not normally distributed (p<10^−13^); however normalcy was established by applying a Box Cox power normalization [33] with λ = 0.2296. Thus a simple formula to capture the distribution of our photosensitivity data is to first draw values from a normal distribution with mean = −4.257 and standard deviation = 0.004674, before applying the transformation (1+data*λ)∧(1/λ). This gives the correct mean and variance for our data, while better capturing the skewness and kurtosis than would a normal distribution.

### The Dynamic Relationship between Bleached Pigment and Receptor Waveguiding

We found a strong inverse association between amount of pigment in the dark and the waveguiding efficiency (photosensitivity) of a cone. We pointed out previously [15] that this echoes bleach-dependent results from psychophysical measurements of the Stiles-Crawford effect [34] and from assessment of cone image quality *ex vivo* [35]. It is possible that a dynamic association exists also – i.e. that cone waveguiding improves as more pigment becomes bleached. We argue that this does in fact occur based on the following analysis.

**Figure 5:**
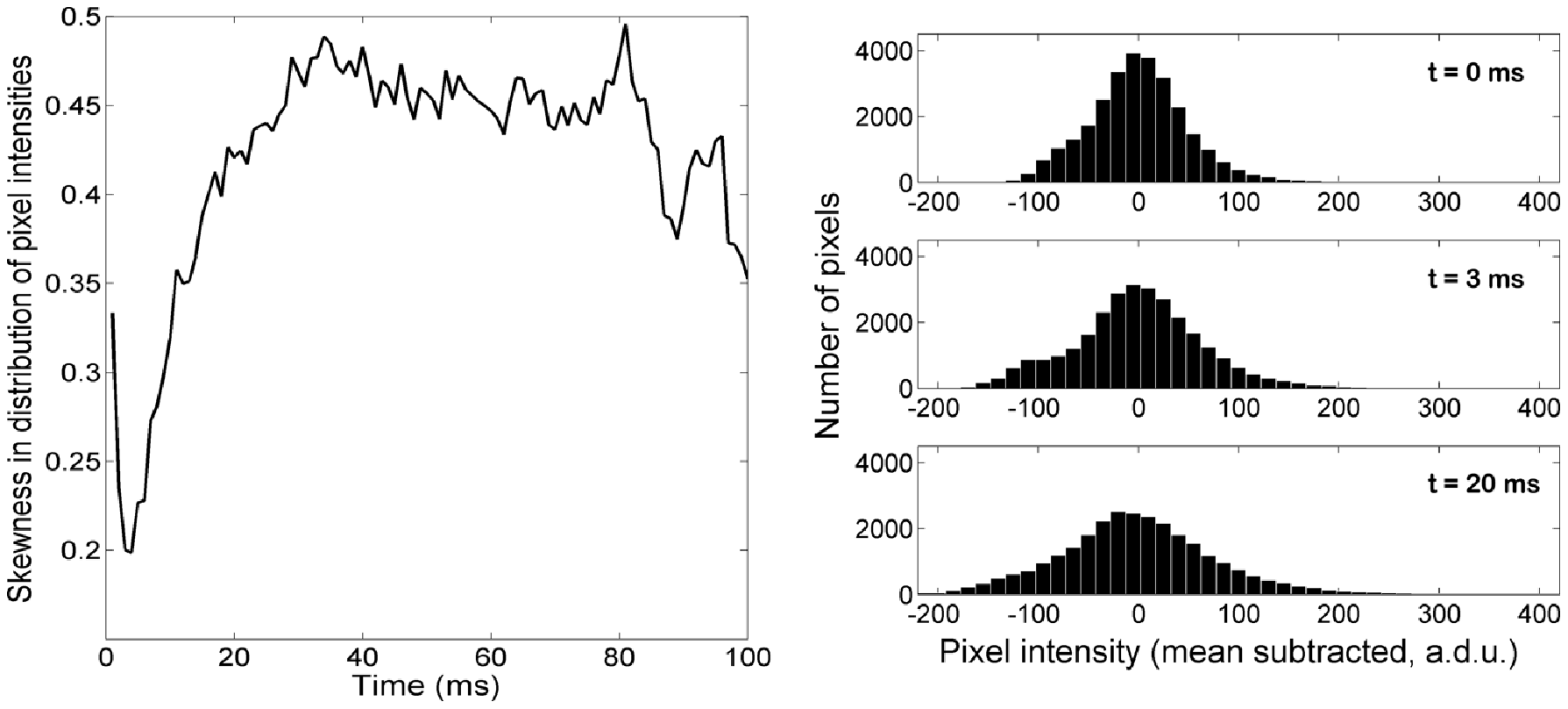
Evolution in cone waveguiding over time. Left: Skewness in the distribution of pixel intensities, as a function of time, for the averaged pseudo-sequence. The minimum occurs at 3 ms. Right: Mean-subtracted histograms from the averaged pseudo-sequence, at t = 0 ms, 3 ms and 20 ms.

If cone waveguiding were held fixed while pigment was bleached, we would expect the large population of pixels that are illuminated by cones to increase in intensity, causing a shift of mass in the image histogram towards brighter intensities. This is expressed as a negative shift in ‘skewness’ (the third moment about the mean, normalized to the variance) of the distribution of pixel intensities. This can be visualized in our data by comparison of the image histogram at t = 0 and t = 3 ms (Figure 5 (right), top and middle respectively). On the other hand, if pigment were held fixed while cone waveguiding was increased, the cones should more closely approximate single mode fibers and hence point sources [36]. Thus the brightest parts in the image would be concentrated into fewer pixels, and a long tail in the image histogram would result, which is expressed as a positive shift in skewness. This can be visualized in our data by comparison of the image histogram at t = 3 and t = 20 ms (Figure 5 (right), middle and bottom respectively). Thus we propose that pigment bleaching and improvements to cone waveguiding have opposing effects on image skewness. A more familiar image statistic to use would be the variance, which is the second moment about the mean, however this does not aid in the separation of these processes since both should act to increase the variance.

Figure 5 (left) plots the average image skewness as a function of time. There is initially a rapid decrease in skewness as the pigment begins to bleach, followed by an abrupt reversal at ∼3 ms, followed by a roughly linear increase in skewness to ∼20 ms. Beyond this point a plateau of sorts is reached, with the final skewness being greater than that in the initial dark-adapted state. The abrupt reversal at 3 ms does not necessarily translate to the abrupt onset of some physiological process, but it does translate to the point at which changes resulting from such a process outweigh those due to bleaching. Thus we submit that the proposed increase in cone waveguiding efficiency begins no later than 3 ms from light onset.

The abrupt reversal in Figure 5 suggests that the improvements in waveguiding are not an immediate consequence of the bleaching of pigment. This is also supported by the work of Walraven [34], who showed that the Stiles-Crawford effect (which is a direct result of receptor waveguiding) recovers around five-fold faster after bleaching than does the pigment itself. An indirect effect of pigment on waveguiding is also supported by work from Hofmann [4], who suggested that light-evoked scattering changes at 800 nm in rod outer segment preparations indicated a fast process in which bleached photopigment initiated structural changes in the phospholipid bilayer itself. Similarly, work from Liebman [37] demonstrated fast (∼5 ms) changes in rod outer segment birefringence in the light, which were also attributed to structural changes in the phospholipid bilayer following structural changes to the pigment. One possible mechanism for these structural changes is the rapid coupling of transducin into the cell membrane following light onset, which has been used to explain light-evoked changes in infrared scatter in isolated rod outer segment fragments [6]. Another possible mechanism is the inactivation of Metarhodopsin II, in which the chromophore is relocated to the ‘exit’ site of its associated opsin. This process has a time constant of ∼3 ms [25], which could conceivably correspond to the reversal shown in Figure 5.

We have considered the following alternate explanations for the sudden reversal noted in Figure 5, but deem them unlikely:

Rapid regeneration of pigment. Since cones themselves undertake some local conversion of 11-cis *retinol* to 11-cis retinal [24], [38], there could be some local cytoplasmic reservoir that can quickly replace bleached chromophore in the opsin. However, it has been shown in the rods that fresh 11-cis retinal cannot enter the opsin binding site until the previous all-trans retinal has left the exit site, which takes on the order of minutes [24]. Thus a millisecond-scale local regeneration in the cones does not seem likely. In addition such rapid regeneration would allow photoproducts to accrue more rapidly, which should cause fluctuations to begin sooner, whereas our results show that fluctuations begin later in cones that contain more pigment.
Formation of heretofore unknown optically active intermediaries from the phototransduction cascade.
Photoreversal of isomerization within a flash, as can occur when flash intensity is extremely high [39]. By comparison to published data (see Fig. 1 of reference [39]), at our calculated flash energy of 4.67×10^5^ Td.s the effect of photoreversal should be negligible.

### The Effect of Dynamic Waveguiding on Derived Cone Parameters

Upon realization that photoreceptor waveguiding efficiency is dynamic, it is necessary to review the validity of our derived optical density and photosensitivity measurements. The simplest possible model is one in which the effect of pigment on waveguiding acts in direct proportion to the instantaneous level of bleached photopigment. In this case the extra “absorptance” that results from compromised waveguiding would decay with the same time course as the “true” absorptance by visual pigment. In that case the decay constant (the photosensitivity) of the two processes would be identical, and our measured photosensitivity would be valid. The dynamic nature of the cone waveguiding would affect only the measurement of pigment optical density, which would be inflated by some fixed proportion with respect to the true optical density.

To within the measurement precision of our data, we find no reason to reject a highly linear relationship between proportion of bleached pigment and cone waveguiding, as evidenced by 77% of cones reaching an R^2^ quality of fit of >0.95 to the simple exponential model. Thus we believe that our measures of photosensitivity are accurate to a reasonable approximation, and that our measures of pigment optical density are robust but are inflated in direct proportion to the true pigment density. This latter problem is actually of little consequence, since we are interested primarily in relative differences between the cones. Truly absolute measures would require manipulation of the entrance and exit pupils of the imaging system in order to discount the effects of stray light [40], which is not a practical solution for high resolution adaptive optics imaging. Thus we believe that the relative comparisons made above (expressed as a coefficient of variation among cones in the imaging field) remain valid. In order to calculate absolute rates of photon capture, previously published studies facilitating absolute measurement of pigment optical density should be incorporated [40], [41].

The apparent linearity between changes to cone waveguiding and bleaching of cone pigment may not hold true beyond the initial bleaching of pigment (for example, see the slow drift of cone B in Figure 2 after the nominal plateau period has been reached). It is hard to say whether such changes would occur under more natural conditions, or are instead some artifact of the cone saturation that undoubtedly results from our very bright imaging light.

### Fluctuations in Cone Intensity

Deviations from the expected rise in cone intensity due to bleaching began by ∼2 ms in some cones, with ∼50% of cones recruited by 7 ms. The extent to which these intensity reversals correspond to the same phenomenon as the later high-frequency fluctuations is difficult to ascertain, due to the confounding influence of the bleach. The latency of the intensity reversals was correlated with the amount of photopigment, suggesting that reversals are light-evoked, however in contrast the later fluctuations did not correlate with any measured bleach parameters. This means that the fluctuations could well occur in a similar form in the dark. We do note however that work by others imaging at 192 fps with 915 nm light showed light-evoked fluctuations at 5–10 ms after light onset, lasting for several hundred milliseconds, with a diffuse frequency spectrum indicative of some non-linear process [17]. These observations are consistent with our own. Non-linearity in the phototransduction cascade is introduced at the stage where cGMP undergoes hydrolysis, since this is dependent on the rate of photoisomerization [25]. Changes in cone circulating current occur within 2 ms of light onset and, for bright flashes, reach a maximum by ∼20 ms [25], [26], a time course which seems consistent with the onset and evolution of intensity reversals seen in our data. Thus a likely explanation for the intensity fluctuations is either the cessation of cone circulating current directly, or associated factors such as membrane polarization or levels of cGMP. The time course of recovery of the cone fluctuation signal was measured to be ∼300–400 ms in other adaptive optics imaging work [17], which also coincides with measurements of cone circulating current [2].

The contrast of fluctuations in the current experiment was dramatically less than seen in our previous imaging paradigm that was essentially identical apart from the slower frame rate [15]. The time between frames in that work was >80 ms, during which time the cone circulating current would have undergone significant recovery [2]. Baylor proposed some time ago that localized, abrupt blockages in the circulating current occur as it attempts to recover [42]; the mechanism of these blockages is not clear, but if they are associated with the formation of scattering boundaries then they could result in more pronounced interference phenomena. Baylor further proposed that blockages in the circulating current are more evident following flashes that bring the photoreceptor closer towards saturation [42]. If these blockages are indeed related to cone intensity fluctuations, we may then expect that fluctuations depend upon both the amount of time that the receptors are allowed to recover in the dark, and upon the initial stimulation strength employed. These factors may explain the marked differences in contrast of cone intensity fluctuations that have been apparent between different research groups [12], [15]–[18]. A scatter phenomenon within the length of the outer segment, as opposed to at the boundaries alone, would also explain why interference effects are seen with light of much shorter coherence than the length of the outer segment [12], [14]–[16], [18].

These considerations in regard to the recovery of circulating current may be confirmed in the future by imaging for small periods at high speed, punctuated by brief pauses of varying lengths (up to several hundred milliseconds for full recovery of cone circulating current). It will also be necessary to acquire data with light of wavelength beyond the visible range, to separate baseline from light-evoked responses. Further confirming evidence could be gained by imaging the functional optical signals of the rods [12], which are known to recover their circulating current over much longer time scales than the cones [9]. The introduction of pauses is also likely to reveal more about the link between cone adaptation state and the dynamic changes seen in waveguiding efficiency. These considerations may aid in the maximization of the light-evoked signal, to improve its sensitivity as a test of cone function.

We note that in the cones measured to contain greater amounts of pigment, the onset of fluctuations was delayed (p<10^−3^). If the theory above concerning blockages is correct, cones containing more pigment may therefore show somewhat reduced tendency to become blocked.

## Conclusions

Using adaptive optics, we have studied the initially dark-adapted human cone photoreceptor mosaic under rapid stimulation and imaging with visible light at 1000 fps. We measured significant variability in pigment density and photosensitivity between neighboring cones, which we propose to be a major source of noise for the visual system. We confirmed an inverse link between pigment density and photosensitivity, including a dynamic relationship in which cone waveguiding efficiency is dramatically enhanced shortly following the bleach of visual pigment. Our measurements indicated rapid optical disturbances in the cones that occur over a similar time course to the cessation of cone circulating current or related physiological events. Comparison with previous work led us to advance a theory whereby the amplitude of cone intensity fluctuations may be maximized when cone circulating current is allowed a brief recovery period.

## Supporting Information

Media S1 **A pseudo-sequence showing the average of corresponding frames of all dark-adapted sequences, cropped so that only pixels that were illuminated in every sequence.**
(AVI)
